# A Novel Chitosan Nanosponge as a Vehicle for Transepidermal Drug Delivery

**DOI:** 10.3390/pharmaceutics13091329

**Published:** 2021-08-25

**Authors:** Jin Sil Lee, Hyeryeon Oh, Sunghyun Kim, Jeung-Hoon Lee, Yong Chul Shin, Won Il Choi

**Affiliations:** 1 Center for Convergence Bioceramic Materials, Convergence R&D Division, Korea Institute of Ceramic Engineering and Technology, 202, Osongsaengmyeong 1-ro, Osong-eup, Heungdeok-gu, Cheongju 28160, Korea; jslee92@kicet.re.kr (J.S.L.); hyeryeon.oh@kicet.re.kr (H.O.); shkim0519@kicet.re.kr (S.K.); 2 School of Materials Science and Engineering, Gwangju Institute of Science and Technology, Cheomdan-gwagiro 123, Buk-gu, Gwangju 61005, Korea; 3 SKINMED Co., Ltd., Daejeon 34028, Korea; jhoon@cnu.ac.kr (J.-H.L.); ycshin@amicogen.com (Y.C.S.); 4 Amicogen Inc., 64 Dongburo 1259, Jinsung, Jinju 52621, Korea

**Keywords:** chitosan, poloxamer, nanosponge, skin permeation, model drug

## Abstract

Transepidermal drug delivery achieves high drug concentrations at the action site and ensures continuous drug delivery and better patient compliance with fewer adverse effects. However, drug delivery through topical application is still limited in terms of drug penetration. Chitosan is a promising enhancer to overcome this constraint, as it can enhance drug diffusion by opening the tight junctions of the stratum corneum. Therefore, here, we developed a novel chitosan nanosponge (CNS) with an optimal ratio and molecular weight of chitosan to improve drug penetration through skin. To prepare the CNS, two types of chitosan (3 and 10 kDa) were each conjugated with poloxamer 407 using para-nitrophenyl chloroformate, and the products were mixed with poloxamer 407 at ratios of 5:5, 8:2, and 10:0. The resulting mixtures were molded to produce flexible soft nanosponges by simple nanoprecipitation. The CNSs were highly stable in biological buffer for four weeks and showed no toxicity in human dermal fibroblasts. The CNSs increased drug permeability through human cadaver skin in a Franz-type diffusion cell, with substantially higher permeability with 3 kDa chitosan at a ratio of 8:2. This suggests the applicability of the novel CNS as a promising carrier for efficient transepidermal drug delivery.

## 1. Introduction

Percutaneous drug delivery has been a major focus of many studies to identify alternative drug administration pathways. Transepidermal delivery offers several potential advantages over other administration methods, including high concentrations of drugs at the site of action, fewer side effects, continuous drug delivery, and better patient compliance [1,2,3,4]. Thus, numerous studies have examined various transepidermal drug delivery systems, including lipid-based vehicles, vesicular carriers, particulate lipid carriers, surfactant-based nanocarriers, transfersomes, and polymer-based nanoparticles [5,6,7,8]. Lin et al. developed lipid-based vesicles to increase the skin permeation of drugs; these vesicles were produced using thin-film hydration, and cationic surfactant-modified ultradeformable liposomes used in the vesicles increased drug release and improved transepidermal flux [9]. Moreover, soft lipid-based nanotransfersomes containing eprosartan mesylate were examined, and optimized nanotransfersomes helped achieve enhanced transepidermal flux through Wistar rat skin [10]. Using triblock copolymer, PLGA-PEG-PLGA nanoparticles were previously investigated to assess their effect on skin permeability. The thermoresponsive activity of the triblock polymer nanoparticles promoted the diffusion of drugs into the stratum corneum [11]. However, most drugs are limited with respect to their diffusion ability through skin. The respective skin barrier functions are due to the stratum corneum, which is primarily composed of an outer layer of 10–15 μm thickness containing keratin, abundant corneocytes, and surrounding multilamellar matrix structures. Corneocytes and intercellular lamellae limit the transepidermal flux of drugs and, thus, restrict pharmacological activity [12,13,14,15].

To overcome the major constraints of transepidermal delivery, chitosan was considered as a promising permeation enhancer candidate [16,17,18]. Chitosan, produced through the deacetylation of chitin, is a biocompatible, biodegradable, bioadhesive, and natural polysaccharide comprising N-acetyl glucosamine and glucosamine with β-1,4 glycoside linkages. Chitosan biodegradability contributes to enzymes that cause hydrolysis of bonds such as glucosamine-*N*-acetyl-glucosamine, glucosamines, and *N*-acetyl-glucosamines. In our body, the degradation of chitosan is caused by lysozyme and bacterial enzymes, especially in the colon [19,20]. Furthermore, chitosan, obtained from crab exoesquelets, has several polar groups such as –OH and –NH2 that can act as electron donors [21]. In detail, chitosan, a cationic polymer containing protonated amine groups in repeat units, carries a positive charge and can increase skin permeability by opening the tight junctions of the stratum corneum [22,23,24]. When chitosan attaches to tight junctions, the positive charge of the amine group elicits F-actin depolymerization and disincorporation of the tight-junction protein ZO-1, leading to the loosening of tight junctions [25,26].

Various delivery systems using chitosan have been studied to enhance drug penetration through the skin [27]. Hasanovic et al. [28] used electrostatic interactions between positively charged amino groups and negatively charged tripolyphosphate (TPP) to produce chitosan TPP (CS-TPP) nanoparticles. Diffusion of a drug through porcine skin was assessed over 48 h using small and large CS-TPP nanoparticles, and the permeation of aciclovir was more effective on large CS-TPP nanoparticles than on smaller ones due to stronger interactions with the cell surface. Furthermore, chitosan-coated liposomes were previously investigated as enhancers of transepidermal delivery of agents for photodynamic therapy. Chitosan-coated liposomes markedly increased the skin permeability of a photosensitizer, indocyanine green, compared to uncoated liposomes [29]. The establishment of a transepidermal drug delivery system has been previously investigated using PLGA and chitosan-coated PLGA nanoparticles loaded with a positively charged hydrophilic drug, donepezil hydrochloride (DP), which resulted in a higher DP accumulation in rat skin compared to uncoated PLGA nanoparticles [30]. However, the optimal molecular weight and proportion of chitosan on nanoparticles for improving drug permeation through the skin barrier has not yet been reported.

Therefore, we developed a novel chitosan nanosponge (CNS) with an optimized molecular weight and proportion of chitosan for an improved skin penetration of drugs. The CNS was prepared using a simple nanoprecipitation method and various ratios of chitosan-poloxamer conjugates using chitosan at different molecular weights and poloxamer 407. The stability of the CNS was assessed by storage in a biological buffer for four weeks. Subsequently, the in vitro cytotoxicity of the CNS to human dermal fibroblasts was examined, and the skin permeability of a model drug administered through the CNS was assessed using human cadaver skin.

## 2. Materials and Methods

### 2.1. Materials

Poloxamer 407, para-nitrophenyl chloroformate (pNPC), triethylamine, and pyridine were obtained from Sigma-Aldrich (St. Louis, MO, USA). Water-soluble chitosan (deacetylation degree: 90%; molecular weight: 3 and 10 kDa) was purchased from Amicogen (Jinju, Republic of Korea). D_2_O, Nile red (NR), and ninhydrin reagent (2% solution) were obtained from Sigma-Aldrich. HyClone deionized water (DIW) and phosphate-buffered saline (PBS) were purchased from GE Healthcare Life Sciences (Marlborough, MA, USA). Human adult dermal fibroblasts (hADF) were purchased from the American Type Culture Collection (Manassas, VA, USA). Fetal bovine serum (FBS), Dulbecco’s modified Eagle medium (DMEM), antibiotic–antimycotic (AA) solution, and trypsin were purchased from Thermo Fisher Scientific (Waltham, MA, USA). A Cell Counting Kit-8 (CCK-8) was obtained from Dojindo Laboratories (Kumamoto, Japan).

### 2.2. Preparation of Chitosan-Conjugated Poloxamers

Chitosan-conjugated poloxamers were prepared using a previously described method [31]. For step 1, to prepare activated poloxamers (pNPC-poloxamer) with pNPC, poloxamer 407 (2 g) was dissolved in 30 mL anhydrous dichloromethane (DCM) and activated by dropwise addition of pNPC (323 mg in DCM) and pyridine (130 μL) at 0 °C. The reaction was allowed to proceed in an argon atmosphere at 25 °C under gentle stirring for 17 h. The solution was filtered through a 0.45 μm polytetrafluoroethylene filter, precipitated in cold diethyl ether (10-fold volume), and centrifuged at 2700× *g* for 5 min. The process was repeated at least three times, and the resulting solution was dried under vacuum.

For step 2, to produce chitosan-poloxamer conjugates, activated poloxamer (300 mg) and triethylamine (30 μL) were added to each type of chitosan (3 and 10 kDa; 900 mg each) dissolved in dimethyl sulfoxide (30 mL). The reaction was allowed to proceed at 25 °C for 12 h, and the product was purified by filtration through a 0.45 μm polytetrafluoroethylene filter and precipitated in cold diethyl ether. The resulting precipitate was dried under vacuum for three days.

For the calculation of the degree of substitution of the pNPC-poloxamers, ^1^H-NMR spectra were recorded using ^1^H-NMR spectroscopy (JEOL JNM-ECX-400P; Akishima, Japan). D_2_O was used as a NMR solvent for individual samples. In addition, the analysis was performed with 1 mg/mL of concentration and 400 MHz of frequency at −20~−60 °C. The substitution degree exceeded 98%, as assessed by analyzing the peaks of the phenyl rings (8.21 ppm) of pNPC and those of the propylene oxide (1.07 ppm) of poloxamers.

### 2.3. Production of the CNS

Using 3 and 10 kDa chitosan, two types of CNS were produced (termed CNS3K and CNS10K, respectively) through simple nanoprecipitation [22]. The chitosan-poloxamer conjugate (10 mg) and poloxamer 407 were dissolved in 1 mL acetone at different ratios of chitosan-poloxamer to poloxamer (5:5, 8:2, and 10:0). The mixture was allowed to react at room temperature under rotary shaking at 560 rpm for 2 h and was added dropwise into 5 mL DIW. To remove acetone, the solution was then placed in a fume hood for 12 h and purified by centrifugation at 2700× *g* for 3 min with an Amicon Ultra-15 centrifugal filter (MWCO 100 kDa; Merck Millipore, Burlington, MA, USA). To produce drug-loaded CNS, NR (0.1 mg) was used as a lipophilic model drug and added to the chitosan-poloxamer conjugate mixture (10 mg). Two types of NR-loaded CNS, termed NR@CNS3K and NR@CNS10K, were produced and purified using the methods described above.

In addition, the amount of unloaded NR was measured using a UV-Vis spectrophotometer at 552 nm, and the analysis was performed at pH 7.0 and 25 °C using the calibration curve ranging from 0 to 100 μg/mL (R^2^ = 0.9979).

The loading content of the drug in CNS was then calculated:

$$Loading\;Contents\;\left(L.C\right)=\frac{amount\;of\;loaded\;drug-amount\;of\;unloaded\;drug}{amount\;of\;\mathrm{CNS}}\;\times \;100$$

and drug loading efficiency of the CNSs was calculated as previously reported [22]:

$$Loading\;Efficiency\;\left(L.E\right)=\frac{amount\;of\;loaded\;drug-amount\;of\;unloaded\;drug}{amount\;of\;loaded\;drug}\;\times \;100$$

Furthermore, the hydrodynamic diameters, polydispersity index (PDI), and zeta potential of the CNSs (10 mg/mL in deionized water) before and after NR loading were measured using an electrophoretic light scattering spectrophotometer at 37 °C (ELS-Z2; Otsuka Electronics Co., Osaka, Japan), which is equipped with a diode light source (638 nm) and a detector with a 165° scattering angle.

### 2.4. Ninhydrin Assay

The amount of chitosan conjugation in the CNS was determined using a ninhydrin reaction [32,33,34]. First, ninhydrin solution was prepared using ninhydrin, hydrindantin, DMSO, and lithium acetate buffer (pH 5.2). This solution was mixed with CNSs at a 1:1 volume ratio to allow the formation of Ruhemann’s purple, after which the tubes were quickly sealed. The tubes were briefly shaken by hand and heated to 85 °C in a water bath. After 30 min, the solution was cooled, absorbance (at 570 nm) was measured using a UV spectrophotometer (Agilent 8453; Agilent, Santa Clara, CA, USA), and chitosan concentrations were calculated using a standard calibration curve.

### 2.5. CNS Stability Analysis

The stability of CNS3K and CNS10K with different chitosan ratios was determined by resuspending the compounds in biological buffer. Lyophilized CNS was resuspended in PBS buffer (pH 7.4) and stored at 37 °C in a shaker incubator (100 rpm). Physicochemical characteristics, including the hydrodynamic diameter and the polydispersity of the CNSs, were assessed over four weeks at predetermined time points [22,35].

### 2.6. In Vitro Cytotoxicity Test

The cytotoxicity of CNS3K and CNS10K was assessed using hADF cells. Cells were seeded in a 96-well plate at 10,000 cells/well and grown in DMEM supplemented with 10% FBS and 1% AA at 37 °C in a humidified 5% CO_2_ atmosphere for 12 h. Subsequently, the medium in each well was replaced with fresh medium containing CNSs (100 and 1000 μg/mL) followed by incubation of the cells at 37 °C for 24 h. After this, the medium was replaced with cell culture medium containing 10-fold diluted CCK-8 solution, and cells were incubated at 37 °C for 2 h. A scanning multiwell spectrophotometer (FL600; Bio-Tek, Winooski, VT, USA) was used to measure absorbance spectra at 450 nm. All analyses were performed using three replicates, and the absorbance relative to that of the control (cells treated only with culture medium) was recorded [36].

### 2.7. Skin Permeation of Drugs through CNSs

The skin permeation effect of the model drug, i.e., NR, using CNS3K and CNS10K with different chitosan ratios, was measured using a Franz-type diffusion cell (FDC-6T; Logan Instruments, Somerset, NJ, USA) and fluorescence microscopy and handled according to guidelines of the Animal Care and Use Committee of Chungbuk National University (approval no. CA-20-27, 1 December 2020). First, to mount a full-thickness human cadaver skin sample (58-year-old male; certified intact back skin) obtained from Hans Biomed Co. (Daejeon, Republic of Korea) in a Franz-type diffusion cell, the skin sample was treated as previously reported [37]. Before rehydration in PBS, the skin was cut into 1.5 × 1.5 cm squares. The skin squares were then mounted in the Franz-type diffusion cell at a diffusional area of 0.636 cm^2^. PBS buffer (pH 7.4) was used as the receptor phase, which was maintained in the receptor chamber at 37 °C under constant stirring. Next, CNSs (NR@CNS3K and NR@CNS10K at 5:5, 8:2, and 10:0 ratios) loaded with 250 μg/mL NR in DIW were applied to the skin surface in the donor chamber. At several time points during 24 h, 0.5 mL receptor solution was replaced with the same volume of the fresh buffer to maintain the total volume. The amount of NR that penetrated the skin was assessed using a fluorophotometer (F-7000; Hitachi, Tokyo, Japan) at 552/636 nm (excitation/emission).

To assess the distribution of NR in the skin, diffusion areas of the skin after application of NR@CNS3K, NR@CNS10K, and NR only were separated and fixed using 4% formalin solution. The samples were then embedded in an optimal cutting temperature compound (Tissue-Teks; Sakura Finetek, Kyoto, Japan), and 20 µm sections produced using cryostat sectioning at −20 °C were then mounted on glass slides. NR abundance in the skin was examined using a fluorescence microscope (TE2000-U; Nikon, Melville, NY, USA) [35,36].

## 3. Results and Discussion

### 3.1. Preparation and Physicochemical Characteristics of CNSs

**Figure 1:**
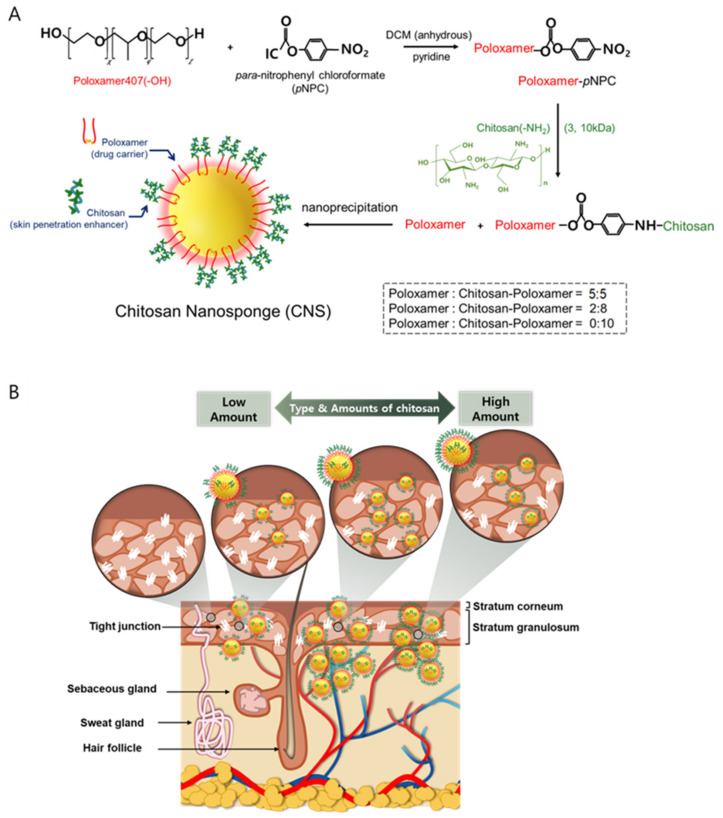
Schematic illustration of (**A**) preparation steps of chitosan-poloxamer conjugates and chitosan nanosponges (CNSs) and (**B**) optimized CNSs for an enhanced skin penetration of drugs according to types and amounts of chitosan, which can open tight junctions in the epidermis.

**Figure 2:**
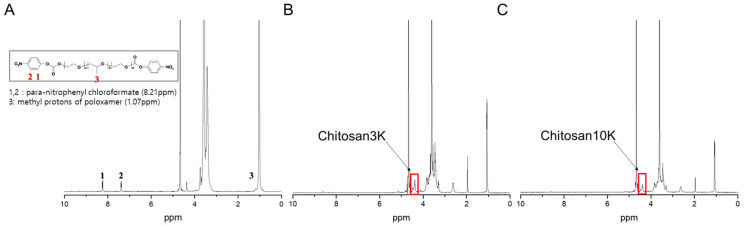
^1^H-NMR spectra of (**A**) p-NPC-poloxamer, (**B**) chitosan3K-poloxamer conjugate, and (**C**) chitosan10K-poloxamer conjugate in D_2_O.

Chitosan-poloxamer conjugates were produced in two steps. In the first step, the activated poloxamer was prepared, and in the second step, chitosan was conjugated to the activated poloxamer. The conjugation of chitosan to the activated poloxamer (pNPC-poloxamer) was achieved through the formation of urethane linkages between the primary amine unit of chitosan and the terminal hydroxyl units of poloxamer in two synthesis steps (Figure 1A). The molecular weight and chitosan proportion of the novel CNS, which was produced using chitosan-poloxamer conjugates, were optimized for the improved penetration of drugs into the skin (Figure 1B). The synthesis of activated poloxamer and chitosan-poloxamer conjugates was confirmed to be above 98%, based on ^1^H-NMR spectroscopy (Figure 2), with which methyl protons (1.07 ppm; 3) of the poly(propylene oxide) block in the poloxamers, the proton in para-nitrophenyl chloroformate (8.21 ppm; 1), and the anomeric protons in chitosan (4.51 ppm) were analyzed [31].

**Figure 3:**
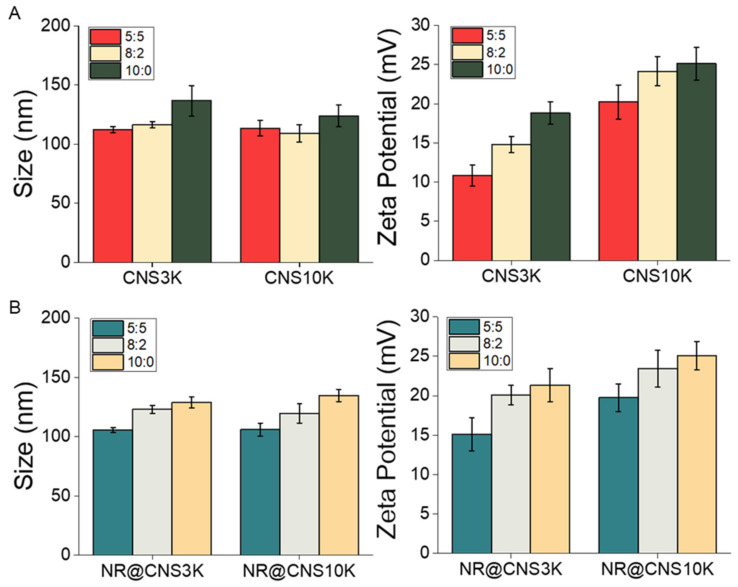
Hydrodynamic diameters and surface charges (zeta potentials) of the chitosan nanosponges (**A**) before and (**B**) after loading with Nile red (NR) at 37 °C (*n* = 3).

CNSs were produced through nanoprecipitation in aqueous solution using different types of chitosan (3 and 10 kDa) and different ratios of chitosan-poloxamer conjugates and poloxamer 407 (5:5, 8:2, and 10:0). Size and surface charges of the CNSs were analyzed using dynamic light scattering, and the CNSs showed monodispersity, with a PDI below 0.3 (Figure S1). Moreover, through TEM analysis, the morphology of the CNSs was determined to be spherical-like, with a similar size to DLS (Figure S2). The diameters and surface charges of CNSs increased slightly with increasing chitosan-poloxamer conjugate to poloxamer ratios due to the increasing amount of chitosan, as previously reported [38]. To assess the physicochemical characteristics of the CNSs before and after drug loading, NR-loaded CNSs (NR@CNS3K and NR@CNS10K) were prepared with a loading amount of ca. 1% (wt.%) and over 95% loading efficiency of NR. There are several methods to separate the free drug from nanoparticles, such as centrifugation, dialysis method, and centrifugal ultrafiltration. Compared to physical separation methodology, ultrafiltration does not need extensive time and dilution. To improve accuracy using the methodology, the minimized range molecular weight cutoff device was used and interaction between nanoparticle and filter materials reduced [39,40]. The characteristics of CNSs after drug loading were similar to those of unloaded CNSs and showed a similar increasing tendency of the size and zeta potential with increasing chitosan ratios (Figure 3B). This suggests that CNSs may be used as carrier vehicles for various lipophilic drugs in the biomedical field.

To enhance the skin permeation of a drug, various amounts of chitosan as an enhancer were incorporated into the CNSs using chitosan-poloxamer conjugates. The amounts of primary amines of chitosan in the CNSs were 15.1%, 24.3%, and 31.3% (wt.%) in CNS3K and 29.5%, 47.4%, and 59.4% (wt.%) in CNS10K, at 5:5, 8:2, and 10:0 ratios, respectively (Table 1). Taken together, the amount of chitosan in CNSs increased with increasing chitosan-poloxamer conjugate proportions and with an increasing molecular weight of chitosan.

### 3.2. CNS Stability

**Figure 4:**
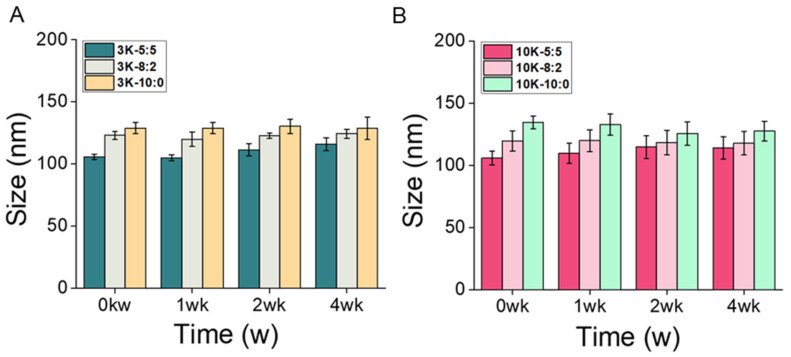
Stability analysis of (**A**) CNS3Ks and (**B**) CNS10Ks over four weeks of storage in phosphate-buffered saline at 37 °C under shaking at 100 rpm.

The stability of CNS3K and CNS10K with different chitosan ratios (5:5, 8:2, and 10:0) was tested using a physiological solution (i.e., PBS; pH 7.4) as reported previously [17,32]. Changes in the hydrodynamic diameters of the CNSs were negligible throughout four weeks of storage in PBS at 37 °C, suggesting an excellent colloidal stability of CNS in a physiological medium (Figure 4).

### 3.3. In Vitro Cytotoxicity of CNS

**Figure 5:**
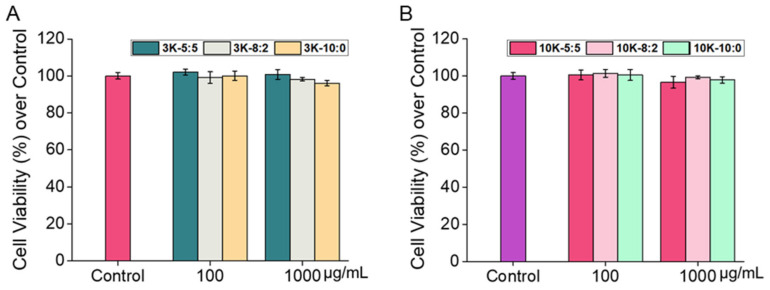
Effects of (**A**) CNS3Ks and (**B**) CNS10Ks on the viability of human dermal fibroblasts as assessed using a CCK-8 assay after incubation at 37 °C for 24 h. Shown are the means ± standard deviation (*n* = 5).

Generally, in vitro cytotoxicity has been studied to predict skin irritability [36]. In the current study, the cytotoxicity of the CNSs (CNS3K at ratios of 5:5, 8:2, and 10:0; CNS10K at ratios of 5:5 and 8:2; and NR@CNS10K at a ratio of 10:0) to hADF was determined. The acute cytotoxicity of the CNSs at 100 and 1000 μg/mL to fibroblasts after 24 h was assessed using a CCK-8 assay, where none of the CNSs exhibited significant cytotoxic effects, resulting in a cell viability of above 95% in each case, regardless of the type and ratio of chitosan (Figure 5). In addition, since the chitosan used in this study was water soluble, the significant cytotoxicity of CNSs did not show at even high concentration compared to a previous study [41]. These results suggest that the CNSs can be used in vivo to assess their efficacy in animal models.

### 3.4. In Vitro Skin Permeation of NR through CNSs

**Figure 6:**
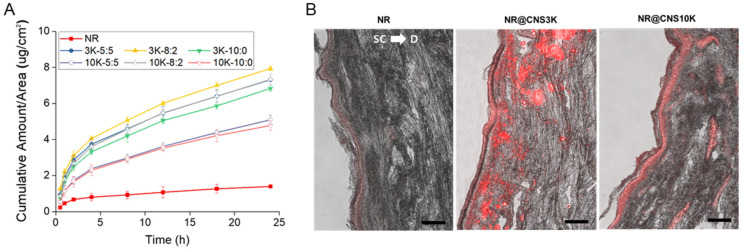
(**A**) In vitro skin permeation of Nile red (NR) through human cadaver skin at several time points over 24 h, with CNS3Ks and CNS10Ks (at ratios of 5:5, 8:2, and 10:0). (**B**) Fluorescence images showing the permeation and distribution of NR in skin samples using CNS at an 8:2 ratio after a 24 h in vitro skin permeation test. The scale bar indicates 100 µm. (SC: stratum corneum and D: dermis).

The transepidermal permeation of NR-loaded CNSs (NR@CNSs) was examined using full-thickness human cadaver skin in a Franz-type diffusion cell, as previously reported [36,37,42]. The skin permeation of NR as a lipophilic model drug was markedly improved by CNSs compared to that of NR without CNS (Figure 6A). As previously reported [43], the cationic charge of chitosan can improve the penetration into stratum corneum and viable skin. The amount of NR with CNSs that permeated through the skin was over five times larger than without CNS. In particular, CNS3K showed a considerably more efficient penetration than CNS10K. This result may have been due to the fact that chitosan with a high molecular weight can inhibit interaction with the layer underneath the skin surface and, subsequently, decrease skin permeability [44]. More interestingly, the optimal ratio of chitosan-poloxamer conjugates and poloxamer for the most efficient skin penetration of drugs was 8:2, regardless of the type of chitosan. Poloxamers are surfactants that might increase flexibility and mechanical strength, leading to an enhanced permeability through an optimized ratio [9,45,46]. These results suggest that the penetration of the lipophilic model drug across the skin barrier was improved using CNSs composed of chitosan as an effective enhancer and poloxamer to improve skin permeability [13,37,45,46].

To visualize the distribution and permeability of NR, skin samples were vertically sectioned and the intensity of red fluorescence of NR in skin treated with NR@CNSs was evaluated; it was found to be higher than that of the NR only group (Figure 6B). As expected, the fluorescence intensity exhibited by skin treated with NR@CNS3K (8:2) was markedly higher compared to that of skin treated with NR@CNS10K (8:2). These results suggest that CNS3K with the 8:2 ratio may be a potential vehicle for the efficient transepidermal delivery of lipophilic drugs.

## 4. Conclusions

We developed a novel CNS with an optimal chitosan type and ratio as an effective enhancer of drug penetration through skin. Although the hydrodynamic diameters and surface charges of the CNSs increased slightly with increasing amounts of chitosan-poloxamer conjugates, the diameter was below 200 nm, and the surface charge was approximately 10 to 25 mV. The physicochemical properties of the CNSs were similar before and after loading of the model drug NR. The CNSs were stable for a substantial period of time (four weeks) under physiological conditions. These conditions did not cause distinct differences in the physicochemical characteristics of the CNSs, and they showed no noticeable cytotoxicity, exhibiting a cell viability of above 90%, to human dermal fibroblasts, at even high concentration of chitosan. Importantly, the skin permeation of the model drug was improved by CNSs, especially by CNS3K (8:2), which showed a markedly increased drug permeation, compared to free model drug. Taken together, our results suggest that the novel CNS prepared in this study may be used as a vehicle for efficient transepidermal drug delivery.

## Figures and Tables

**Table 1:** Amount of chitosan in chitosan nanosponges (CNS3Ks and CNS10Ks) with different ratios of chitosan as analyzed using a ninhydrin assay.

Types of CNS	Amount of Chitosan (wt.%)		
	5:5	8:2	10:0
CNS3K	15.1 ± 2.7	24.3 ± 3.7	31.3 ± 2.1
CNS10K	29.5 ± 2.2	47.4 ± 2.9	59.4 ± 4.2

## Data Availability

Not applicable.

## Supplementary Materials

The following are available online at https://www.mdpi.com/article/10.3390/pharmaceutics13091329/s1, Figure S1. Polydispersity index (PDI) and distribution of (A) CNS3K and (B) CNS10K with different chitosan-poloxamer conjugate to poloxamer ratios. Figure S2. TEM images of (A) CNS3K and (B) CNS10K.
