# Supplementary material for: A Novel Chitosan Nanosponge as a Vehicle for Transepidermal Drug Delivery
Source: Pharmaceutics. 2021 Aug 25;13(9):1329. doi: 10.3390/pharmaceutics13091329 (PMC8468160; doi:10.3390/pharmaceutics13091329)
Supplement: Supplementary file 1 [file pharmaceutics-13-01329-s001.zip › pharmaceutics-1313355-supplementary.pdf]

pharmaceutics

Supplementary Material: A Novel Chitosan Nanosponge as a Vehicle for Transepidermal Drug Delivery

Jin Sil Lee, Hyeryeon Oh, Sunghyun Kim, Jeung-Hoon Lee, Yong Chul Shin and Won Il Choi

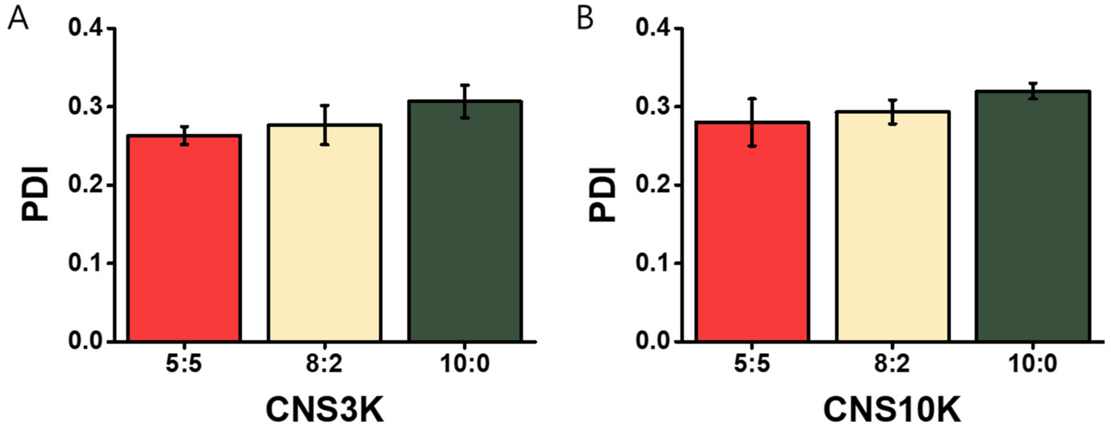

Figure S1. Polydispersity index (PDI) of (A) CNS3K and (B) CNS10K with different chitosan-poloxamer conjugate to poloxamer ratios.

Morphlogy analysis of CNS. For the analysis of the morphology of CNSs, the CNS3K and CNS10K with an optimized ratio (8 :2) were dropped onto a TEM grid with a 200-mesh carbon film and air-dried for 2 days. Then, they were observed by transmission electron microscopy (TEM; JEM-2100Plus, JEOL, Tokyo, Japan). As shown in Figure S2, the morphlogies of CNS3K and CNS10K were spherical-like, and the diameters of CNSs were around 100 to 150 nm, similar to those measured by DLS.

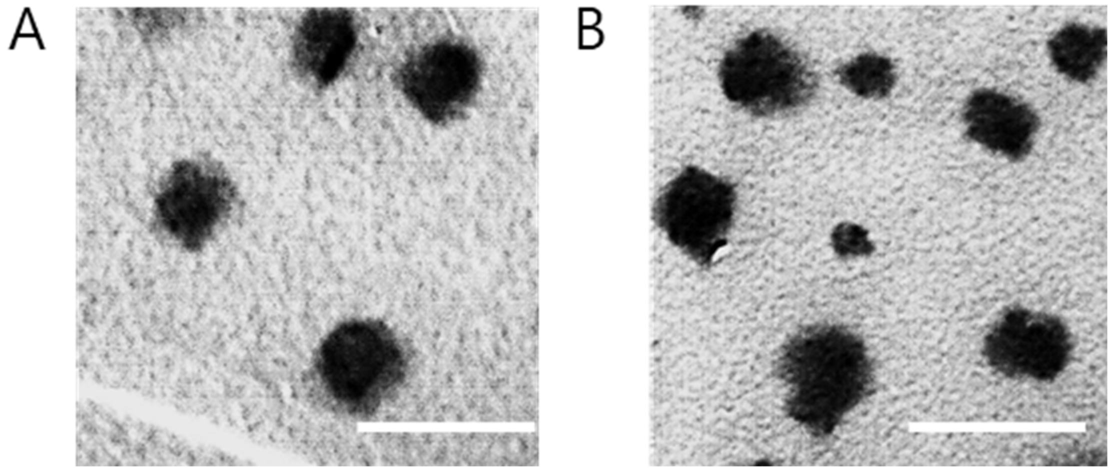

Figure S2. TEM images of (A) CNS3K and (B) CNS10K. The scale bar indicates 200 nm.
